# Multistage delivery of CDs-DOX/ICG-loaded liposome for highly penetration and effective chemo-photothermal combination therapy

**DOI:** 10.1080/10717544.2018.1482975

**Published:** 2018-11-20

**Authors:** Xiao Xue, Ting Fang, Luyao Yin, Jianqi Jiang, Yunpeng He, Yinghui Dai, Dongkai Wang

**Affiliations:** a Department of Pharmaceutics, School of Pharmacy, Shenyang Pharmaceutical University, Shenyang, P.R. China;; b Department of Traditional Chinese Medicine, School of Pharmacy, Shenyang Pharmaceutical University, Shenyang, P.R. China

**Keywords:** Carbon dots, doxorubicin, indocyanine green, liposome, highly penetration, chemo-photothermal

## Abstract

Nanoparticles (NPs) have proven to be effective drug carriers in diagnosis and therapy of cancer. But, they faced a contradictory issue that NPs with large size appear weak tumor penetration, meanwhile small size resulted in poor tumor retention. Herein, we fabricated doxorubicin conjugated carbon dots (CDs-DOX) and indocyanine green (ICG)-loaded liposomes (ICG-LPs) named CDs-ICG-LPs using a modified reverse phase evaporation process, and with high incorporation in the aqueous core. The CDs-ICG-LPs exhibited good monodispersity, excellent fluorescence/size stability, and consistent spectra characteristics compared with free ICG or DOX. Moreover, the CDs-ICG-LPs showed higher temperature response, faster DOX release under laser irradiation. In the meantime, the fluorescence of DOX and ICG in CDs-ICG-LPs was also visualized for the process of subcellular location *in vitro*. In comparison with chemo or photothermal treatment alone, the combined treatment of CDs-ICG-LPs with laser irradiation synergistically induced the apoptosis and death of DOX-sensitive HepG2 cells. *In vivo* antitumor activities demonstrated CDs-ICG-LPs could reach higher antitumor activity compared with CDs-DOX and ICG-LPs for H22 tumor cells, and suppressed H22 tumor growth *in vivo*. Notably, no systemic toxicity occurrence was observed after repeated dose of CDs-ICG-LPs with laser irradiation. Hence, the well-defined CDs-ICG-LPs exhibited great potential in targeting cancer imaging and chemo-photothermal therapy.

## 1. Introduction

Cancer is regarded as one of the most challenging threatens due to the high morbidity and mortality. Nanoparticles (NPs)-based drug delivery has gained mounting attention in diagnosis and therapy of cancer. Up to now, plenty of nanomedicines have been approved. Commonly, nanomedicines could passively accumulate in tumor by virtue of the enhanced permeability and retention (EPR) effect (Yasuhiro & Maeda, 1986; Saxena et al., 2003; Gao et al., 2016; Maeda et al., 2016; Wang et al., 2017). However, the multiplicity of barriers to effective transport will limit the integral optimization of delivery for such systems. When administered intravenously, nano-sized agents tend to circulate for a long period of time if they are not small enough to be excreted by the kidney or large enough to be rapidly recognized and trapped by the reticuloendothelial system (RES) (Kobayashi and Brechbiel, 2005). And, they faced a contradictory issue in delivering drugs into tumors: NPs with large size were characterized with weak tumor penetration, meanwhile NPs with small size resulted in poor tumor retention (Hu et al., 2015). Therefore, it is essential to develop novel NPs that could adapt the tumor microenvironment and improve the homogenous distribution of drugs. To solve this problem, we proposed a multistage drug delivery system using liposome enclose carbon dots (CDs) in the aqueous center. Furthermore, indocyanine green (ICG) is inserted into the bilayer membrane which could intelligently liberated CDs from large size in the presence of photothermal function (Liang et al., 2014; Sercombe et al., 2015).

Recent years have seen increased activities in developing multifunctional nanomaterials that can enable bioimaging, disease detection, and drug delivery simultaneously (Liang et al., 2014; Maldiney et al., 2014; Wolfbeis, 2015; Yang et al., 2015). One may first differentiate between two kinds of fluorescent imaging. The first involves imaging based on fluorescent chemical agents (such as rhodamine). The second covers methods for imaging of samples or cells that have been made fluorescent using synthetic fluorescent probes, labels, or NPs (Reina et al., 2015; Zheng et al., 2015). Carbon dots (CDs), a new class of carbon-based nanomaterial, has received considerable attention due to its fascinating optical properties, unique surface activity, as well as excellent biocompatibility appear to be promising candidates for drug/gene delivery carrier (Zheng et al., 2014; Ding et al., 2015; Yang et al., 2015; Feng et al., 2016; Yuan et al., 2017). Furthermore, the average size of CDs would be smaller than 10 nm, which might achieve fairly high tumor permeability in the course of drug delivery. Previously, we reported the development of CDs functionalized with a nuclear localization signal-carbon dots (NLS-CDs) for cell nucleus imaging. In the architecture, DOX was covalently conjugated to NLS-CDs through a pH-sensitive hydrazone bond using hydrazinobenzoic acid as a linker. The results indicated that the NLS-CDs may server as a prospective candidate for nucleus targeted drug delivery (Yang et al., 2015).

ICG is a near-infrared (NIR) contrast agent commonly used for *in vivo* cardiovascular and eye imaging. It is the only NIR fluorescent dye approved by the U.S. Food and Drug Administration and the European Medicines Agency for human use (Food & Drug Administration, 2013). With its 820 nm NIR emission wavelength, ICG is considered a good *in vivo* contrast agent with minimal interference from blood and tissue autofluorescence (∼500–600 nm) (Nguyen & Tsien, 2013). More interestingly, ICG is a photothermal agent that strongly absorbs NIR light and converts it into cytotoxic heat for tumor treatment (Shirata et al., 2017).

In aqueous environments, ICG molecules aggregate and ICG fluorescence readily degrades (Saxena et al., 2003). In blood, ICG binds to plasma proteins, enhancing its fluorescence intensity. To overcome the protein-binding dependency, we attempted to add ICG to liposomes. ICG can physically interact with phospholipids in the liposome membrane and modify the stability and quantum yield of ICG as well as the structure and stability of the lipid membrane (Kraft & Ho, 2014). As the ICG turns absorbed light energy into heat, heat is transported to the surrounding lipid bilayer and the lipids undergo a phase transition followed by CDs-DOX release. Therefore, the multistage system could benefit from its large size for better retention effect in tumor and then release small size to contribute to better penetration efficiency. In addition, combination therapy has been considered as a promising strategy to improve therapeutic efficiency and minimize side effects (Sun et al., 2011; Mauceri et al., 1998).

In this study, we designed liposome packaging nucleus-targeted CDs in order to achieve high retention and penetrability in tumor. As a chemotherapy drug that acts on DNA inside the nucleus, DOX was covalently conjugated to NLS-CDs through a pH-sensitive hydrazone bond using hydrazinobenzoic acid as a linker. Meanwhile, ICG can physically interact with phospholipids in the liposome membrane, which could modify the stability and quantum yield of ICG as well as receive chemotherapy and photothermal therapy simultaneously. In addition to these, we have showed that ICG was able to cause phase transition in liposomes under steady near infrared light and control release of CDs-DOX in tumor. Here, we reveal a multistage delivery system that can carry, release over time, and deliver two types of NPs into tumor cells. The use of this system is envisioned to open new avenues for avoiding biological barriers and delivering more than one therapeutic agent to the target at a time, in a time-controlled fashion.

## 2. Materials and methods

### 2.1. Materials

Doxorubicin hydrochloride (DOX·HCl) was purchased from T&W Group (Shanghai, China). 4-hydrazinobenzoic acid (HBA) was obtained from Adamas (Shanghai, China). 1-ethyl-3-[3-dimethylaminopropyl] carbodiimide hydrochloride (EDC) and *N*-hydroxysuccinimide (NHS) were supplied by Aladdin Reagent Co., Ltd. (Shanghai, China). ICG was purchased from Xiya Reagent (Shandong, China). Egg phosphatidylglycerol (EPG) and 1,2-distearoyl-sn-glycero-3-phosphoethanolamine-*N*-methoxy-polyethylene glycol-2000 (DSPE-mPEG2000) were purchased from Shanghai Advanced Vehicle Technology Pharmaceutical Co., Ltd. (China). Cholesterol was obtained from Sinopharm Chemical Reagent Co., Ltd. (Shanghai, China). Annexin V-FITC apoptosis detection kit was purchased from Beyotime Institute of Biotechnology (Jiangsu, China). 3-(4, 5-dimethylthialzol-2-yl)-2,5-diphenyltetrazolium bromide (MTT), dimethyl sulfoxide (DMSO), 4′,6-diamidino-2-phenylindole (DAPI), acridine orange (AO), and ethidium bromide (EB) were purchased from Sigma-Aldrich (St. Louis, MO). Anti-Ki 67 primary antibody and Cleaved Caspase-3 (CC3) were obtained from Wanleibio (Shenyang, China). All chemicals and reagents were of analytical grade and used without further purification.

### 2.2. Synthesis of NLS-CDs-HBA-DOX (CDs-DOX) complexes

The NLS-CDs were prepared as described in our previous studies (Yang et al., 2015). CDs-DOX complexes were constructed according to a reported protocol with slight modifications. Briefly, 0.5 mmol HBA was dispersed in 50 mL of pH 7.4 phosphate buffer solution (PBS), and 1 mmol EDC and 1 mmol NHS were then added to the solution. Afterward, the reaction mixture was stirred at room temperature for 4 h to activate HBA. Subsequently, 20 mL of NLS-CDs solution (20 mg/mL) was added to the mixture and the reaction was carried out at room temperature for 48 h. Finally, the solution was dialyzed against deionized water using a cellulose dialysis membrane (molecular weight cutoff: 1000 Da) for 48 h to remove unreacted reagents. The solid powder of HBA-modified NLS-CDs (HBA-CDs-NLS) was obtained by freeze-drying. Then, the antitumor drug DOX was conjugated to the hydrazine group of HBA-CDs-NLS through hydrazone bond. And, 0.20 g of HBA-CDs-NLS was dissolved in 20 mL of DMSO, and 5 mL of DOX stock solution (10 mg/mL in DMSO) was added under stirring, followed by the addition of a drop of acetic acid. The reaction was performed for 72 h, thereafter, the resulting solution was dialyzed against water for 48 h and the solution inside the dialysis membrane was lyophilized to gain the CDs-DOX. All these procedures were carried out in the dark.

### 2.3. Preparation of CDs-ICG-LPs

The control CDs-DOX-liposomes (CDs-LPs), ICG-liposomes (ICG-LPs), and CDs-ICG-LPs were prepared by reverse-phase evaporation method (Bealle et al., 2012; Szoka & Papahadjopoulos, 1978). SPC, cholesterol, EPG, DSPE-mPEG2000, and ICG were dissolved in chloroform in a 50:10:1:1:0.5 molecular ratio. Afterward, CDs-DOX dispersed in 3 mL PBS was introduced before sonication at room temperature for 20 min to produce a water-in-oil emulsion. Preparation was immediately transferred to a round-bottom flask, and organic solvents were evaporated with a rotary evaporator at 30 °C until the gel phase disappeared. Followed by brief vortex mixing and then continued evaporation at normal temperatures and pressures, until a homogeneous suspension was obtained. Then, the dispersion was extruded through a 400-nm polycarbonate membrane using a mini-extruder (Avanti Polar Lipids, Alabaster, AL). The process was repeated through 200 and 100 nm membranes. The CDs-LPs and ICG-LPs were prepared using the same method without addition of ICG or CDs-DOX. ICG self-quenching was reduced, and the density of ICG in the lipid membrane was optimized.

### 2.4. Characterization of CDs-ICG-LPs

The average diameter, zeta potential, and size distribution of CDs-ICG-LPs and CDs-DOX were determined by dynamic light scattering (Zetasizer Nano ZS, Malvern Instruments, UK). The morphologic examination was further performed by transmission electron microscope (TEM) with negative stain method. Before analysis, the test samples were placed on a carbon-coated copper grid, and stained with 1% (w/v) phosphotungstic acid and air-dried prior to TEM observation. The absorption and fluorescence spectrum of ICG and CDs-DOX were determined using UV-Vis spectrometer (UV-Probe-1700, Shimazu, Japan) and multi-function plate reader (Varioskan Flash, Thermo Fisher Scientific, Waltham, MA).

The encapsulation efficiencies (EE) of ICG and CDs-DOX in formulations were both determined using Sephadex G-100 microcolumn-centrifuging. After the Sephadex G-100 microcolumn was established, the addition of 0.2 mL CDs-ICG-LPs to the top of the microcolumn, the column was centrifuged at 900 × g for 3 min and the separated liquid was collected. Then, 0.2 mL pH 7.4 buffer was added and the column was centrifuged using the same method to elute the liposomes. The above process was repeated twice, and the eluent was collected. Methanol was used to dilute the eluent to a volume of 10 mL to calculate the concentration encapsulated inside the liposomes (C_EN_). Another 0.2 mL of liposomes was directly diluted to a volume of 10 mL, but it was not subjected to microcolumn centrifugation, and sample determined as total concentration (C_Total_). Methanol was used to break the liposomes and dilute to a volume of 10 mL prior to sample determination to calculate encapsulated inside the liposomes. Then, the concentration of ICG and CDs-DOX was measured using UV-Vis spectrometer at *λ*_max_ of 784 and 480 nm, respectively. The EE of ICG and CDs-DOX was calculated according to the following formula.

$$\text{EE}\left(\%\right)={\text{C}}_{\text{EN}}/{\text{C}}_{\text{Total}}\times 100\%$$

### 2.5. Photostability of CDs-ICG-LPs

The photostability was assessed using determining the fluorescence emission spectra of free ICG, CDs-DOX, and CDs-ICG-LPs at predetermined times. The fluorescence spectra were examined with a fluorescence spectrometer (F900, Edinburgh Instruments Ltd., UK) at 365 or 740 nm excitation wavelength, 5 nm excitation slit. The photostability test was carried on under the condition of 25 °C and 4500 lx for 5 d.

### 2.6. Temperature evaluation induced by NIR laser irradiation

In this study, 0.2 mL of PBS containing free ICG, ICG-LPs or CDs-ICG-LPs (ICG concentration: 25 μg/mL) was added into 96-well plates. The PBS was used as a blank control. A NIR laser equipment (Changchun New Industries Optoelectronics Tech Co., Ltd., China) emitting 808 nm laser at a power density of 2 W/cm^2^ was used to irradiate these samples for 5 min. The temperature changes of each group were recorded by an infrared thermal imaging camera (LaserSight, Optris, Germany).

### *2.7. In vitro *DOX release from CDs or CDs-ICG-LPs

The release profiles of DOX from CDs-DOX or CDs-ICG-LPs complexes were investigated by the dialysis method in pH 7.4 and 5.5 PBS (Yang et al., 2015; Mo et al., 2013; Moku et al., 2016), respectively. In brief, the complexes (with/without laser irradiated) were dispersed in 5 mL of PBS and then were sealed in a dialysis bag. Afterward, the dialysis bag was immersed in 45 mL of PBS at 37 °C with continuous shock. At arranged time intervals, 5 mL of the solution outside the dialysis bag was taken and replaced with the same amount of fresh PBS. The percentage of released DOX was quantified using a UV-Vis spectrophotometer at 480 nm.

### *2.8. In vitro *cytotoxicity

The cytotoxicity of ICG, CDs-DOX, and CDs-ICG-LPs complexes against HepG2 cells was assessed by MTT method. HepG2 cells were seeded in 96-well plates at a density of 1 × 10^4^ cells/well and incubated for 24 h. Then, the culture medium was removed and fresh medium containing free DOX, free ICG, CDs-DOX or CDs-ICG-LPs complexes was added to the well, respectively. After 48 h of incubation, 20 μL of MTT solution (5 mg/mL) was added and further incubated for 4 h. Subsequently, the medium was thrown away and 200 μL of DMSO was added to disperse the formazan precipitate, and the multi-function plate reader (Varioskan Flash, Thermo Fisher Scientific) was used to measure optical density of the solution at 570 nm. The HepG2 cells incubated with ICG and CDs-ICG-LPs for 24 h were exposed to 2 W/cm^2^ 808 nm laser irradiation for 5 min, and the cells were incubated for another 4 h to investigate the photothermal influence on cytotoxicity.

### 2.9. Cellular uptake

The cellular uptake of free DOX, free ICG, CDs-DOX or CDs-ICG-LPs complexes was determined by flow cytometry. HepG2 cells were grown overnight at a density of 2 × 10^5^ cells per well in six-well plates. And, cells were exposed to each sample for different times (1, 2, and 4 h) and with/without laser irradiated, then the cells were washed twice with PBS and centrifuged at 2000 rpm for 5 min to gather cells. Finally, cells were resuspended in PBS, and the uptake of DOX was quantified using a FACSCalibur flow cytometer (BD Biosciences, Franklin Lakes, NJ).

### 2.10. Confocal microscopy

HepG2 cells (2 × 10^5^ cells/well) were seeded into six-well chambered plates in 2 mL of medium, respectively. After 24 h, the medium was replaced by the medium containing CDs-DOX or CDs-ICG-LPs complexes (with/without laser irradiated) for 4 h. After the incubation, cells were washed thrice with PBS and fixed with 4% of paraformaldehyde, followed by addition of DAPI for cell nuclei staining. Finally, cells were washed three times and sealed onto object slide. The intracellular localization of CDs, DOX, and ICG was observed by confocal laser scanning microscopy (CLSM) (Nikon C2+, Japan).

### 2.11. AO/EB staining and flow cytometry analysis of apoptosis

Cell morphology of free DOX, CDs-DOX or CDs-ICG-LPs complexes-induced apoptosis was researched by AO/EB double staining. HepG2 cells were embedded in 24-well plates and incubated for 24 h; afterward, the medium was discarded and replaced with fresh medium containing various concentrations of each sample and incubated for another 24 h. Then, the cells were stained with 2 μL of AO/EB mixture (100 μg/mL) for 5 min, and the images of the treated cells were collected by an inverted fluorescence microscope (Olympus IX71, Japan).

The percentage of apoptotic cells was quantified by flow cytometry using apoptosis detection kit. Briefly, HepG2 cells grown in six-well plates were treated with DOX, CDs-DOX or CDs-ICG-LPs for 24 h, then cells were washed and suspended in binding buffer followed by staining with 5 µL of Annexin V-FITC and 10 µL of propidium iodide (PI) for 15 min in the dark. The samples were then determined by flow cytometry.

### 2.12. Animals and tumor model

Male Kunming (KM) mice (6–8 weeks) were obtained from Shenyang Pharmaceutical University Experimental Animal Center (Shenyang, China). The animal studies were carried on in accordance with the guidelines of the Experimental Animal Center and ratified by the Animal Ethical Committee of Shenyang Pharmaceutical University. *In vivo* antitumor activity of DOX, CDs-DOX, ICG-LPs, or CDs-ICG-LPs complexes was assessed in KM mice implanted sarcoma H22 cells. H22 cells (1 × 10^7^/mL) were injected subcutaneously into the right anterior armpit of KM mice.

### *2.13. In vivo *imaging and biodistribution

The imaging studies were carried out when tumor volumes of mice reached to about 200 mm^3^. The mice were randomly divided into three groups and intravenously injected with CDs-DOX, CDs-ICG-LPs with laser irradiation or CDs-ICG-LPs without laser irradiation, respectively. The equivalent ICG dose was kept at 5 mg/kg. The images of CDs and ICG in mice were taken at 1, 2, 4, 6, 8, and 24 h after injection using the *ex*/*in vivo* imaging system (FX PRO, Carestream Health, Toronto, Canada) with 410 and 760 nm as excitation wavelength, and 530 and 810 nm filter to collect the FL signals. The mice of CDs-ICG-LPs with laser irradiation group were irradiated after injection 1 and 4 h. After injection at 24 h, the mice were sacrificed, and organs including heart, liver, spleen, lung, kidney, and tumors were collected for the *ex vivo* imaging under the same conditions as above.

### *2.14.In vivo *antitumor evaluation

When tumors were 200–300 mm^3^ in size, the tumor-bearing mice were randomly divided into five groups (six mice each group) and were injected intravenously via the tail vein with normal saline, free DOX, CDs-DOX, ICG-LPs or CDs-ICG-LPs complexes (at a dose of DOX 5 mg/kg and ICG 5 mg/kg body weight), respectively. After injection 4 and 8h, the tumors of mice were irradiated by the 808 nm laser for 5 min. The mice receiving saline with laser irradiation was defined as the control. The bodyweight changing of each mouse was recorded every 2 d for 15 d. On the 15th day, mice were sacrificed and the tumor tissues were harvested to measure the tumor weight. The inhibition rate of tumor growth was calculated using the below equation:

$$\text{IR}\%=\frac{W_{\text{c}}-W_{\text{t}}}{W_{\text{c}}}\times 100$$

where *W*_c_ and *W*_t_ mean the tumor weight of control saline group and test group.

The rest of the mice was used to measure the survival curves. The survival time of all mice was recorded each day until all the mice’s death.

### 2.15. H&E staining, immunohistochemistry, and immunofluorescence

The pretreated mice were sacrificed by standard decapitation, the major organs and tumors were harvested immediately, rinsed with PBS, fixed with formalin and embedded in paraffin. Five micrometer sections were cut with a paraffin slicing machine and were dewaxed to water using xylene and different concentration of alcohol. In all cases, hematoxylineosin (H&E) dyes were conducted.

Subsequently, tumor slides were then treated with immunohistochemical reactions. In order to analyze the proliferation status of the tumor tissue, Ki-67 immunohistochemistry (IHC) was carried out assessed with anti-Ki-67 primary antibody. In order to evaluate the apoptosis level of tumor tissue, serial sections were reacted with CC3 specific antibody diluted 1:100, which could recognize large fragments of activated caspase-3. All the experiments were performed according to manufacture of different IHC kits (Saxena et al., 2003; Saxena et al., 2004).

### 2.16. Statistical analysis

All data are shown as mean ± SD unless otherwise indicated. ANOVA was used for statistical analysis among groups by SPSS software (New York, NY). The differences were defined as significant for *p* < .05 and very significant for *p* < .01.

## 3. Results and discussion

### 3.1. Preparation and characterization of CDs-DOX and CDs-ICG-LPs

**Scheme 1.**
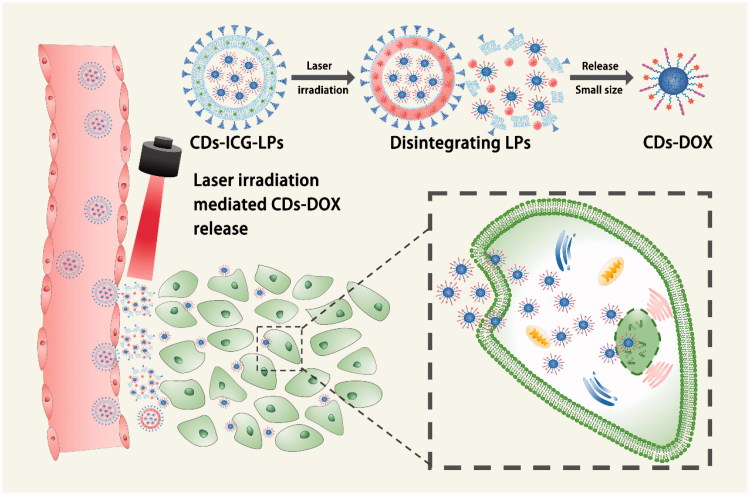
Schematic illustration of the CDs-ICG-LPs accumulating at the tumor site by the EPR effect and releasing CDs-DOX by laser irradiation DOX.

**Figure 1.**
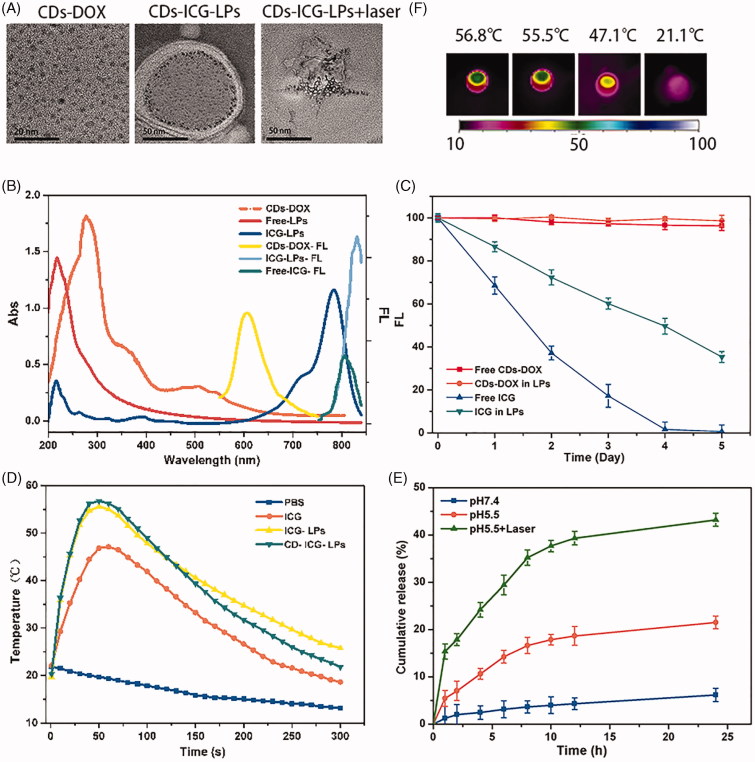
Morphology and characterization of CDs-ICG-LPs. (A) TEM images of CDs-DOX and CDs-ICG-LPs with or without continuous laser irradiation. After laser irradiation CDs-ICG-LPs were broken up into smaller pieces with heat-induced disruption. (B) Absorption and fluorescence spectrum of ICG, CDs-DOX, and CDs-ICG-LPs. (C) FL stability of free ICG, CDs-DOX, and in CDs-ICG-LPs. (D) Maximum temperature profiles of free ICG, ICG-LPs, CDs-ICG-LPs, and PBS as a function of the irradiation time under continuous laser irradiation at a power intensity of 2 W/cm^2^. (E) DOX release profiles from CDs-ICG-LPs with and without laser irradiation at pH 7.4 or pH 5.5. The data are shown as mean. It was consistent with the *in vitro* release experiments that the DOX release of CDs-ICG-LPs could be accelerated by laser irradiation. (F) Infrared thermographic maps of centrifuge tubes with CDs-ICG-LPs, CDs-DOX, ICG-LPs, or PBS were measured at 5 min with an infrared thermal imaging camera after continuous laser irradiation.

The synthesized structure is described in Scheme 1. The CDs-ICG-LPs were assembled from CDs-DOX, ICG, SPC, EPG, and DSPE-PEG through a reverse-phase evaporation method. The TEM image (Figure 1(A)) verified the formation of CDs-DOX and liposomes with well-defined spherical shape and homogeneously distributed. The surface potential of CDs-DOX and CDs-ICG-LPs was measured by dynamic light scattering (DLS). The zeta potential of CDs-DOX and CDs-ICG-LPs was between +32.6 mV and −12.9 mV. The average diameter of CDs-DOX and CDs-ICG-LPs was 3.8 and 87.4 nm respectively with a suitable particle size and distribution for targeted drug delivery and long circulation (Hu et al., 2015; Blanco et al., 2015; MacEwan & Chilkoti, 2017). And, 99% of CDs-ICG-LPs in number distribution were all less than 100 nm, which will be suitable for passive tumor targeting of drug delivery through the EPR effect. The drug encapsulation efficiency (EE) and drug-loading efficiency (LE) of the NPs are crucial for their clinical application. Supporting Information shows the EE or LE of the drug of NPs formulations. The EE or LE of DOX was observed at 47.9% or 1.87%, and the EE or LE of ICG was 89.3% or 1.68% for CDs-ICG-LPs. The stability of CDs-ICG-LPs dispersed in the PBS solution was examined and the results are shown in Table S1. We can see that CDs-ICG-LPs had good stability at low temperatures, but there was a slight leakage of CDs-DOX at 25 °C in a month, so we propose to store CDs-ICG-LPs at low temperature. The particle sizes of CDs-ICG-LPs remained around the initial particle size without aggregation and precipitation over 1 month, suggesting a great stability of CDs-DOX and ICG.

**Figure 2.**
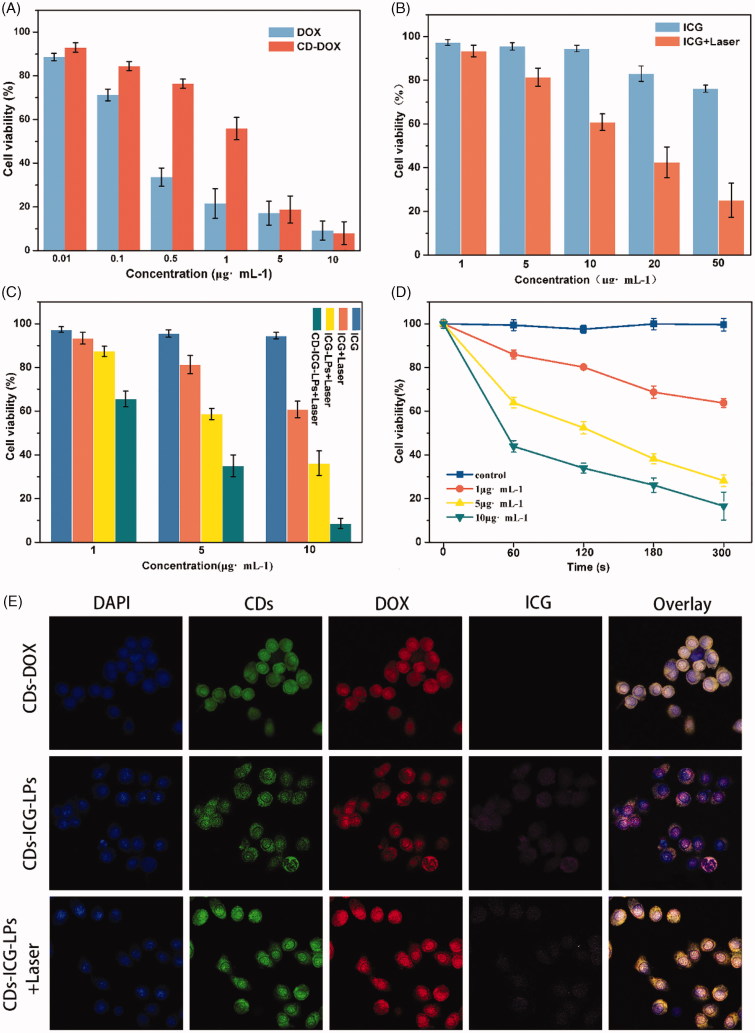
The chemo and photothermal treatments in HepG2 cells. (A) Quantitative evaluation of cell survival for HepG2 cells treated with free DOX and CDs-DOX for 48 h. (B) Quantitative evaluation of cell survival for HepG2 cells treated with free ICG or ICG plus laser irradiation. (C) Survival of HepG2 cells versus free ICG, ICG-LPs, or CDs-ICG-LPs plus laser irradiation. (D) Survival of HepG2 cells versus different ICG concentration in LPs and laser irradiation time. (E) Confocal microscope images of HepG2 cells incubated with CDs-DOX and CDs-ICG-LPs for 4 h with or without laser irradiation. The cell nuclei were stained with DAPI and the co-localization was confirmed by the intensity overlay of the fluorescent signals.

The absorption or FL spectra of encapsulated CDs-DOX and ICG are shown in Figure 1(B). Without entrapped in liposome, free ICG would quickly aggregate and sedimentate. Compared with CDs-ICG-LPs, the emission peak intensity of free ICG was only 28.34%. The absorption or emission peak of ICG in CDs-ICG-LPs was red-shifted from 1 to 778 nm or 21 to 835 nm. Absorption and emission peak corresponding CDs-DOX consistent with the spectra of free DOX were located at 480 and 605 nm. There are also no severe fluorescence changes of the CDs-ICG-LPs under 25 °C and 4500 lx over 5 d, while the fluorescence intensity of free ICG in Figure 1(C) almost completely degraded after 4 d. However, the fluorescence intensity of ICG in LPs drops to 50% by the fourth day and still maintains 30% of its initial value on the fifth day. These data indicated that both the ICG and CDs-DOX maintained their optical properties after encapsulation with CDs-ICG-LPs.

ICG is an amphiphilic molecule consisting of a lipophilic polyaromatic polyene group and a hydrophilic sulfonate group. Previous report has revealed free ICG can be degraded in aqueous solution due to the saturation of the double bonds in the conjugated chain, resulting in a simultaneous decline of fluorescence and absorption. And, free ICG in aqueous solution would aggregate to form dimers and oligomers, resulting in fluorescence self-quenching. Liposomal formulations can be used to incorporate ICG molecules in the membrane in monomeric form, because the lipophilic part of ICG is inserted into the phospholipid bilayer, whereas the hydrophilic part faces the aqueous environment. Therefore, we can infer that liposome bilayer keeps ICG as monomeric status which could actively contributes to enhance fluorescence, stability and photothermal effect of ICG. The protective effect of liposomes seemed to be due to the lipid molecule which protected the entrapped ICG by isolating it from aggregate and the surrounding environment (Saxena et al., 2004).

To evaluate the photothermal potency of CDs-ICG-LPs, we monitored the temperature changes under laser irradiation *in vitro* using an infrared thermal imaging camera. With the laser irradiation at 2 W/cm^2^ for 5 min, the temperature of CDs-ICG-LPs, ICG-LPs, and free ICG maximally increased to 56.8, 55.5, and 47.1 °C, while the PBS did not increased (Figure 1(D)). Such a variation of CDs-ICG-LPs, ICG-LPs, and free ICG could increase temperature over 50 °C, leading to an irreversible damage to tumor cells (Saxena et al., 2004; Lim et al., 2008). CDs-ICG-LPs and ICG-LPs formulation had a higher temperature response than free ICG under laser irradiation, similar to that of previously reported ICG encapsulated lipid NPs which has more efficient laser-dependent temperature increase than free ICG (Zheng et al., 2012). The probable reasons were that ICG entrapped in CDs-ICG-LPs or ICG-LPs had higher inspissated concentration than free ICG, and the excitation thermal radiation was also entrapped in the enclosure of LPs, generating higher energy efficiency and lower heat dissipation in the CDs-ICG-LPs or ICG-LPs after laser irradiation. The infrared thermographics showed that the maximum temperature of CDs-ICG-LPs, ICG-LPs, free ICG, and PBS at 5 min after laser irradiation (Figure 1(F)). Additionally, the release behavior of DOX from the CDs-ICG-LPs complexes was investigated at pH 7.4 and 5.5, respectively. Moreover, laser irradiation accelerated DOX releasing from the CDs-ICG-LPs, as shown in Figure 1(E); the LPs without laser irradiation showed DOX release of 6.18% at pH 7.4 and the total release reaching 21.51% at pH 5.5 by 24 h. The accelerated release of DOX can be illuminated by cleavage of the hydrazone bond under the mildly acidic conditions, which forecasted that the CDs-DOX complexes could control release the drugs in the acidic intracellular compartments. And, the laser irradiation observably increased the release rate of DOX from LPs to 24.19% over the first 4 h. After laser irradiation, the total release of DOX by 24 h was significantly enhanced to 43.22%, indicating that the drug release of LPs could be controllable by laser irradiation. We further studied the morphology of LPs irradiated by 2 W/cm^2^ 808 nm laser for 5 min using TEM. Figure 1(A) shows that LPs were broken up into smaller pieces with heat-induced disruption. It was consistent with the *in vitro* release experiments that the DOX release of LPs could be accelerated by laser irradiation and had pH-stimulate respond ability at the same time.

**Figure 3.**
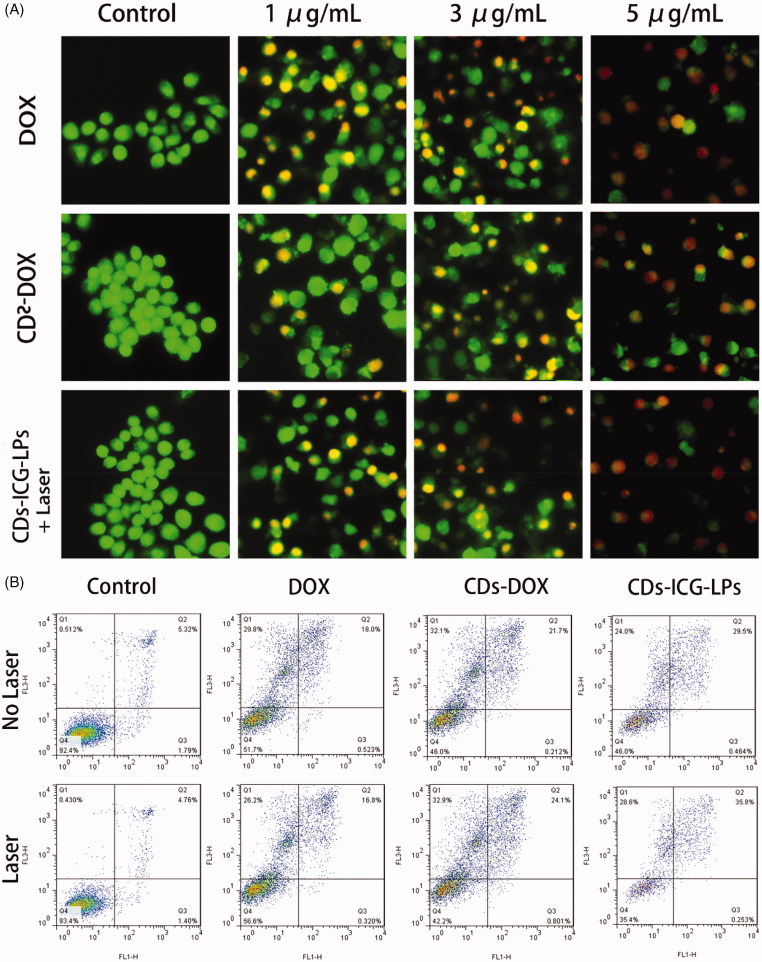
(A) AO/EB staining of apoptotic HepG2 cells incubated with free DOX, CDs-DOX, and laser irradiated CDs-ICG-LPs for 24 h. (B) Flow cytometric analysis of apoptotic/necrotic cells in HepG2 cells treated with free DOX, CDs-DOX, and CDs-ICG-LPs for 24 h, untreated cells were used as a control and photothermal was evaluated as well.

### 3.2. Intracellular uptake

The cellular uptake of CDs-DOX and CDs-ICG-LPs was analyzed by flow cytometry and visualized by CLSM, separately. Figure 2 shows the flow cytometry data of HepG2 cells treated with CDs-DOX and CDs-ICG-LPs for the referential times. It is definite to see that the CDs-DOX group displayed higher cellular uptake when compared with free DOX, and the final amount of cells treated with CDs-DOX was 1.2-fold that of DOX after 4 h incubation. This can be attributed to the difference in cellular uptake mechanism (Li et al., 2014). It is common knowledge that free DOX is transferred into cells by passive diffusion, whereas previous studies have demonstrated that the cellular uptake of CDs-DOX was through caveolae and clathrin-mediated endocytosis as well as passive diffusion, so the uptake of CDs-DOX was significantly increased due to the cooperation of the two pathways. Interestingly, CDs-ICG-LPs with laser radiation significantly increased the uptake rate of DOX in HepG2 cells compare to without laser group, indicating enhanced internalization of drugs by LPs formulation (Figure 2).

CLSM was performed to further investigate the cell uptake behavior and subcellular localization of CDs-DOX and CDs-ICG-LPs in HepG2 cells. As shown in Figure 2(E), the CDs-DOX was localized in both nucleus and cytoplasm after 4** **h of incubation, and predominantly to the nucleus location. The detail was confirmed by monitoring co-localization with nuclear dye DAPI, which suggested that CDs could deliver their cargo into the nuclei. Whereas the CDs-DOX entrapped CDs-ICG-LPs without laser radiation showed a slight cellular and nuclear accumulation, and the signal of DOX and CDs displayed weaker overlap with DAPI. The possible reason could be that hydrophilic PEG coated on the LPs can form a hydrated outer shell, thereby protecting the CDs-ICG-LPs from being lightly up taken by cells. On the other hand, the negative surface charge of CDs-ICG-LPs would have electrostatic repulsion with cytomembrane of HepG2 cells, which prevented the LPs from traversing cytoplasm to nuclear. And, this consequence as well consistent with the results of flow cytometry (Figure S2).

Notably, CDs-ICG-LPs formulation with laser radiation not only significantly increased the FL signals of DOX and CDs in HepG2 cells, but also facilitated them entry into the nuclei (Figure 2(E)). These phenomena would be result from the laser-caused hyperthermia, which could disrupt CDs-ICG-LPs into smaller pieces and help CDs-DOX release from LPs fast. Also, the heat caused by laser radiation could enhance cell permeability and fluidity, therefore increased the drug accumulation inside cancer cells and nuclei (Tang et al., 2010). Taking these factors into consideration, it is not surprising that hyperthermia significantly increased the uptake of CDs and DOX in HepG2 cells. After incubation for 4 h, both CDs and DOX exhibited markedly enhanced fluorescence intensity in HepG2 cells, indicating the efficient cellular uptake of the complexes, which was consistent with the results obtained by flow cytometry.

### 3.3. Cell viability studies

The cell viability of HepG2 cells incubated with blank liposomes, free DOX, free ICG, CDs-DOX, or CDs-ICG-LPs was shown in Figure S1 and Figure 2. From Figure S1, we conclude that liposomes showed slight cytotoxicity and good biocompatibility. After 48 h incubation, it can be seen that the survival rate of HepG2 cells in CDs-DOX group was over 55% at low concentrations (0.01–1 μg/mL). Similar trend in cytotoxicity was also observed in free DOX group, but the cell viability reduced to 20% at a concentration of 1 μg/mL and decreased further with increasing the dose of DOX (Figure 2(A)). The cell viability of CDs-DOX group was much higher than that of free DOX at the dose of 0.5 and 1 μg/mL, which indicated that the complexes could reduce the cytotoxicity of DOX and this may be ascribed to the slow release rate of DOX from the complexes (Figure 2(A)). Moreover, a considerable reduction in cell viability was observed at high concentrations of CDs-DOX (5–10 μg/mL) due to the increasing total cumulative dose of DOX.

We further measurably investigated the effect of irradiation time and ICG concentration in liposomes on cell survival at 2 W/cm^2^ 808 nm (Figure 2(B,C)). Without irradiation, treatment of LPs containing different amount of ICG slightly affects growth of HepG2 cells, suggesting their well biocompatibility (Figure 2(B)). After 5 min laser irradiation, the cell viability of HepG2 cells treated with LPs containing ICG was 73.26% and 57.5% under 5 μg/mL and 10 μg/mL, respectively. While treated with LPs containing 50 μg/mL ICG, the cell viability of HepG2 cells was significantly decreased to 25.0% after 5 min laser irradiation (Figure 2(C)). Free ICG was of relatively lower cytotoxicity compared with the LPs containing the same concentration of ICG (Figure 2(C)). The viability of HepG2 cells under different ICG concentration appeared similar declining trend under different laser irradiation time (Figure 2(B)). As a control, laser irradiation alone had almost no impact on the cell viability.

As expected from the quantitative results, the chemo-photothermal treatments led to extremely cytotoxicity than chemo or photothermal treatment alone (Figure 2(C)). Delightedly, significantly lower viabilities were observed when the HepG2 cells incubated with CDs-ICG-LPs complexes and irradiated by 2 W/cm^2^ 808 nm laser for 5 min. The cell viability at 24 h of HepG2 cells treated with ICG-LPs containing 5 μg/mL ICG plus laser irradiation was 50.13%, and the survival of cells treated with CDs-DOX containing 0.5 μg/mL DOX was 70.98%. However, when the cells were treated with CDs-ICG-LPs containing ICG and DOX with the same concentrations plus laser irradiation, the survival of cells was prominently reduced to 32.89%. With the treatments of CDs-ICG-LPs with 0.5 μg/mL DOX and 1, 5, or 10 μg/mL ICG plus laser radiation, the survival of HepG2 cells at 24 h was 65.52%, 32.89%, and 4.17%, respectively. Quantitatively, the cell viability significantly decreased with the increase of concentrations of ICG-LPs. The combination of CDs-ICG-LPs and laser irradiation led to more effective cytotoxicity than either treatment alone in HepG2 cultures. The results were in accordance with previous observation that CDs-ICG-LPs with laser radiation enhanced the uptake of CDs-DOX effectively in cancer cells.

### 3.4. Cell apoptosis analysis

To investigate the effect of CDs-DOX and CDs-ICG-LPs on cell apoptosis in HepG2 cells, AO/EB staining as well as Annexin V-FITC/PI assay was carried out to visualize the changes in cell morphology and quantify the rate of apoptosis, respectively.

#### 3.4.1. Observation of cell morphology

Dual AO/EB fluorescent staining, visualized under a fluorescent microscope, was performed to identify apoptosis-associated changes of cell morphological during the process of apoptosis. It speculated that AO penetrated normal and early apoptotic cells with intact membranes, emitting green fluorescence when bound to DNA. While EB only entered cells with damaged membranes, such as late apoptotic and dead cells, exhibiting orange-red fluorescence when bound to concentrated DNA fragments or apoptotic bodies (Venkatesan et al., 2013; Liu et al., 2015). Therefore, the AO/EB staining method achieved the detection of apoptosis and can distinguish between late apoptotic and dead cells.

Figure 3(A) represents the AO/EB-stained HepG2 cells treated with diverse concentrations of free DOX, CDs-DOX, and CDs-ICG-LPs for 24 h. In the negative control group, cells showed uniform bright green nuclei with integrated cell membrane, while cells copied with free DOX, CDs-DOX, or CDs-ICG-LPs showed obvious morphological changes, such as chromatin condensation and nuclear fragmentation. Early apoptotic cells with yellow-green nuclei and fragmented chromatin were observed in the presence of 1 μg/mL of CDs-DOX, while further increasing the concentration to 3 μg/mL, cells displayed condensed and asymmetrical chromatin and appeared orange to red, which is characteristic of late apoptotic cells. With increasing concentrations (5 μg/mL), necrotic cells were seen, with red nuclear EB staining and no nuclear condensation.

The results revealed that treatment of HepG2 cells with CDs-ICG-LPs plus laser irradiation induced similar apoptosis in a dose-dependent manner compare with CDs-DOX, which also can be seen in the free DOX group. Whereas compared with DOX-CDs, a noticeable increase in the number of necrotic cells was found at the higher dose of free DOX and CDs-ICG-LPs, and this may be attributed to the sustained release behaviors of CDs-DOX complexes and reduced the toxicity of high concentration of free DOX. On the other side, the laser-caused hyperthermia could disrupt CDs-ICG-LPs into smaller pieces and enhanced cell permeability and fluidity; therefore, it increased the drug cytotoxicity. The cells appeared to be in the process of severe disintegrating and fragmented.

#### 3.4.2. Apoptosis analysis by flow cytometry

Annexin V-FITC/PI staining in combination with flow cytometry is commonly used to quantitatively distinguish the cell apoptosis, which were sorted into necrotic cells (Q1, Annexin V-FITC negative, PI positive), late apoptotic cells (Q2, both Annexin V-FITC and PI positive), early apoptotic cells (Q3, Annexin V-FITC positive, PI negative), and intact cells (Q4, both Annexin V-FITC and PI negative) (Chen et al., 2008). As shown in Figure 3(B) and Figure S3, after 24 h treatment, the apoptotic ratio of untreated HepG2 cells was 7.11%, the fraction of apoptotic cells for free DOX was 18.52%; however, the proportion of necrosis was as high as 29.80%. Fortunately, CDs-DOX displayed an enhanced rate of apoptosis (24.70%) and lower percentage of necrotic cells (16.50%) in comparison with the free drug, which is beneficial for improving the therapeutic effect and reducing the toxicity of DOX. CDs-ICG-LPs without laser irradiation appeared comparatively weaker apoptosis (19.12%) and necrotic cells (10.10%). While irradiated with 2 W/cm^2^ 808 nm for 5 min, the apoptotic and dead percentage of CDs-ICG-LPs reached the maximum at 24.90% and 32.90%. This result was consistent with the observation of AO/EB staining. Taken together, we conclude that the CDs-ICG-LPs complexes may be excellent candidates for drug controlled release with enhanced antitumor activity via the apoptosis pathway.

### *3.5. In vivo *imaging and biodistribution

**Figure 4.**
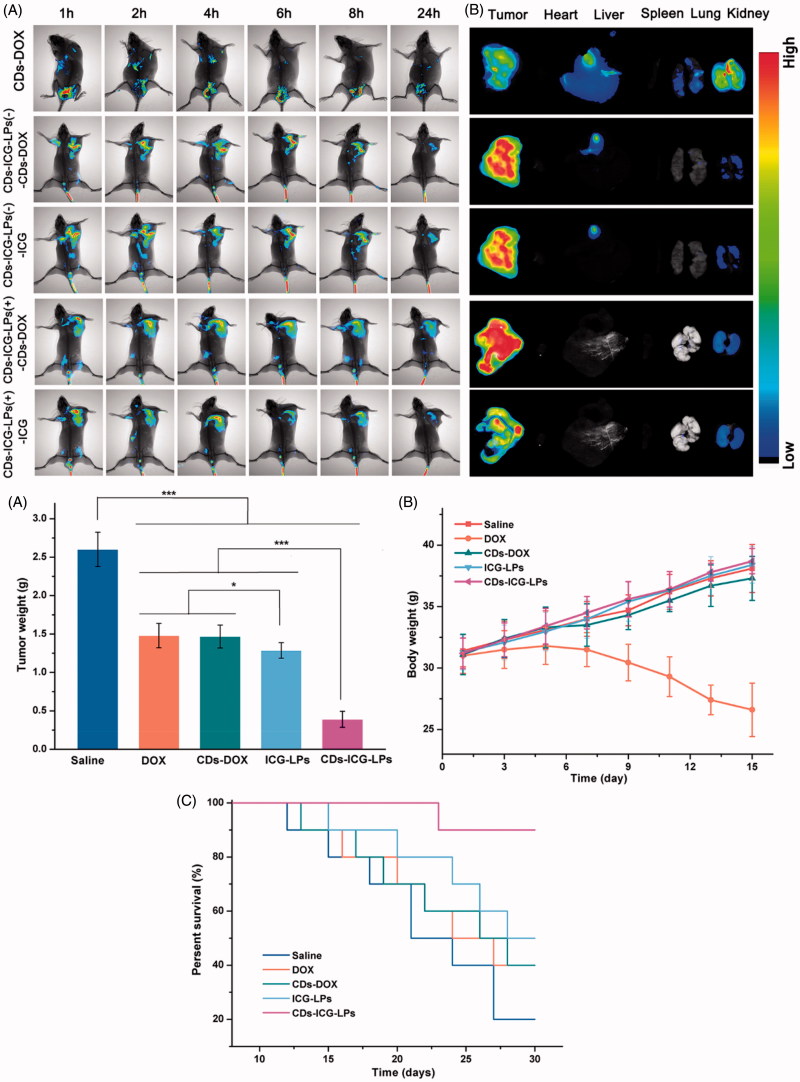
*In vivo* imaging of CDs-ICG-LPs compared with CDs-DOX as controls delivered systemically via tail vein injections in mice implanted with H22 cell. (A) CDs-DOX, CDs-ICG-LPs *in vivo* images taken at 1, 2, 4, 6, 8, and 24 h time point (‘–’ and ‘+’ mean treated with/without laser irradiation). (B) Fluorescence images of organs and tumors in tumor-bearing mice after 24 h post-injection of CDs-DOX or CDs-ICG-LPs. The mean tumor weight (C), mice body weight (D), and survival time (E) of mice bearing H22 tumor treated with free DOX, CDs-DOX, ICG-LPs, CDs-ICG-LPs and mice given saline as a control in different groups.

The *ex*/*in vivo* imaging system was used to evaluate the tumor targeting and *in vivo* distribution of CDs-ICG-LPs in KM mice. Figure 4(A,B) shows the fluorescence signal and intensity distributions at different time point for CDs-DOX, CDs-ICG-LPs delivered systemically via tail vein injections. It showed that the fluorescence signals of CDs-DOX were primarily located in the kidney after injection. Obvious fluorescent signals was also detected in the tumor at first 4 h, and then revealed a gradual decrease of fluorescence signals in the tumors and no evident fluorescence signals in the tumors after 24 h injection. In 8 h post-injection, ICG and CDs-DOX fluorescence signals of CDs-ICG-LPs were almost located around the tumors all the time and little in other organs. At 24 h time point post-injection, ICG and CDs-DOX of CDs-ICG-LPs still maintained significant fluorescence signal in the tumor then. Obviously, the tumor distribution was greatly different from CDs-DOX group. CDs-ICG-LPs clearly contrast the tumor with surrounding tissues, which was forceful evidence of the high efficiency of targeting tumor of these NPs. Because of the size in nanoscale, the NPs-based drug carrier ‘CDs-ICG-LPs’ could enter into the tumor vasculature and interstitium by EPR effect. Previous research shows that free ICG was taken up exclusively by hepatic parenchymal cells where it was then secreted into the bile. This clearance pathway was consistent with the observations of initial hepatic localization and eventual total clearance through the biliary tree with minimal acute renal involvement (Zheng et al., 2012). The PEGylated CDs-ICG-LPs appeared specific tumor targeting and greatly extended circulation time for the encapsulated ICG and CDs-DOX. From Figure 4(A), we can see that CDs-ICG-LPs after laser irradiation could achieve effective release of CDs-DOX, which manifested as fluorescence enhance of CDs-DOX and weak of ICG. It is also showed the consistent *ex vivo* distribution and fluorescence intensity of isolated organs in Figure 4(B). The fluorescence performed higher intensity and more widely spread in the tumor of CDs-ICG-LPs after laser irradiation compare with no laser irradiation group. The bigger size made CDs-ICG-LPs could be retained around tumor vasculature and smaller size made CDs-DOX could diffuse into the core of tumor. These initial data provided strong evidence that CDs-ICG-LPs were sufficient for *in vivo* imaging to provide long circulation time, specific tumor targeting and effective control of release by photothermal therapy.

### *3.6.* In vivo *antitumor activities*

Based on the favorable performance of inducing apoptosis, we are interested to performing *in vivo* therapeutic efficacy study to assess the potential of different CDs-DOX or ICG-loaded preparations for inhibiting tumor growth. As shown in Figure 4(C), compared with control saline group, DOX, CDs-DOX and ICG-LPs group appeared significantly (*p* < .01) *in vivo* antitumor activity with the inhibition rate of 43.0%, 43.6% and 50.5%, demonstrating DOX or ICG could inhibit the tumor growth by chemotherapy or photothermal therapy. In comparison, CDs-ICG-LPs group showed an excellent highest antitumor activities among all drug-treated groups (inhibition rate of 85.1%), achieving almost complete tumor regression. This result was probably attributed to the synergistic effect of PEG chain induced long circulation time, combined therapy of DOX and ICG and nuclear targeting of CDs-DOX. The CDs-ICG-LPs could simultaneously deliver CDs-DOX and ICG into tumor sites via EPR effect, which combined chemotherapy with photothermal therapy efficiently. Then, NIR laser irradiated to the tumor, the CDs-ICG-LPs will release CDs-DOX in a moment and CDs-DOX could target the tumor cell nucleus and play an effective cytotoxic effect. This result was in alignment with the trend of our previously studies that the CDs-DOX could penetrate into the tumor nucleus achieving enhanced antitumor activity.

Body weight of different groups was also measured during this period as shown in Figure 4(D). Significantly decrease could be observed for DOX group, suggesting intravenous administration of DOX would cause nonspecific toxicity. However, there was no significant decrease in body weight for saline and other drug-treated groups, indicating the CDs and liposomes based drug delivery system could reduce the systematic toxicity and reach higher therapeutic effect.

From the results above, we could find the CDs-ICG-LPs showed excellent *in vivo* antitumor activities against liver solid tumor, we are also interested to investigate the survival time of different drug-treated groups using Kaplan–Meier survival curve. As shown in Figure 4(E), similar results could be observed as the tumor inhibition study. CDs-ICG-LPs group showed the longest survival time among all groups. This result demonstrated CDs-ICG-LPs showed excellent *in vivo* antitumor effect against tumor tissue and increase the survival time.

### 3.7. H&E staining and IHC

**Figure 5.**
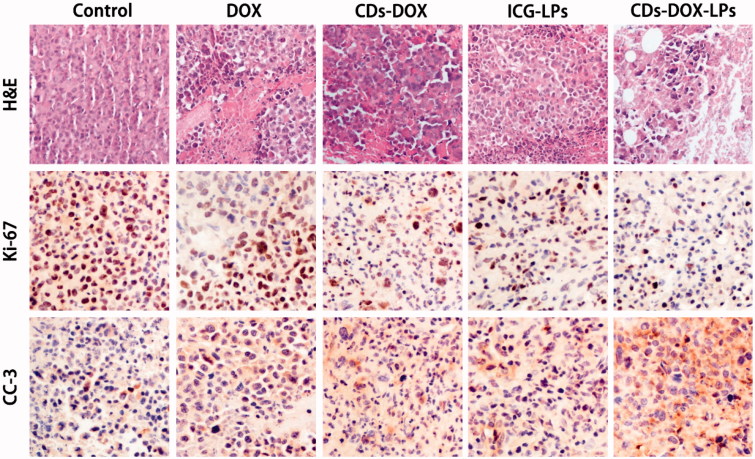
Morphological evaluation in H&E sections of tumor sites (first panel). *In vivo* evaluation of tumor proliferation level by Ki-67 immunohistochemistry (second panel) (proliferation cells shown brown pixel dots). Evaluation of the apoptosis behavior of different formulations by CC3 immunohistochemistry (third panel) (brown pixel in cytoplasm represents the apoptosis level).

From previous experiment about *in vivo* assay, CDs-ICG-LPs complexes could efficiently deliver to tumor sites and inhibit the tumor growth. Besides, we also performed further assays of tumor in order to observe detailed state of the cancer cells after treating with different formulations. Tumor sections were harvested and experimented with H&E staining and IHC. As shown in Figure 5, the H&E staining histological aspects of control group were homogeneity and integrity, which indicated that the tumor was in normal growth and proliferation state. In contrast, the tumor section of DOX, CDs-DOX, ICG-LPs, and CDs-ICG-LPs emerges different levels of inflammation and cell necrosis, which appeared as inhomogeneous dyeing and messy cell morphology. Surprisingly, tumor treated with CDs-ICG-LPs plus laser irradiation present lots of voids and sparse cell distribution, that was probably because the loss of plenty dead tumor cells.

Measurement of proliferative activity is important in determining the tumor grade, recurrence span, and malignancy. So as to quantitatively analysis anti-proliferation effect, tumor tissues were also examined by IHC staining with proliferating marker, Ki-67. Ki-67 is a nuclear markers used to demonstrate the proliferative phase of the cell cycle. As shown in Figure 8, brown in cell nucleus represents the expression of Ki-67. Control group appeared a high expression level of Ki-67, indicating tumor tissues kept a high proliferation level. DOX, CDs-DOX, and ICG-LPs groups showed a lower expression of Ki-67, suggesting DOX or ICG could inhibit tumor proliferation. CDs-ICG-LPs group showed the lightest brown, which illuminating the highest anti-proliferation effect among all groups. It demonstrated that photothermal effectively enhanced cytotoxicity of CDs-DOX and reach grislier decrease to the cell proliferation.

In order to appraise the apoptosis behavior in histological level, CC3 was also used in this section. Advances in the understanding of the molecular events in apoptosis have led to the realization that caspase activation is a specific indicator of this cell suicide mechanism. One of these is that present on activated caspase 3, a ubiquitously distributed caspase that is a main effector caspase of the apoptotic cascade within cells (Gown & Willingham, 2002). For immunohistochemical stains, immunoreactive cells were visualized with DAB chromogen followed by a hematoxylin counterstain. The antibody specific for activated caspase-3 selectively labeled the cytoplasm of cells that had a morphology consistent with apoptosis, as well as the cytoplasm of some morphologically healthy-looking cells (Duan et al., 2003). As intuitively shown in Figure 5, the DOX, CDs-DOX, ICG-LPs, and CDs-ICG-LPs groups appeared increasing brown staining in cytoplasm, which represent the apoptosis were going to be more serious gradually. Nearly, no brown fluorescence could be observed in control groups, suggesting cancerous cells were all kept in a high proliferation status. By quantitative analysis of optical density, CDs-DOX and ICG-LPs slightly improved OD value compared with free DOX. Further, CDs-ICG-LPs group showed the strongest brown stain intensity and highest OD value, suggesting this drug delivery complex could significantly induce cell apoptosis. This result was consistent with the trend observed in Ki-67 and H&E staining. All these histological assays demonstrated the combination of chemotherapy and photothermal therapy drug delivery of DOX by CDs-ICG-LPs could effectively decrease the cell proliferation and reach higher therapeutic effect with promoted apoptosis.

### *3.8.In vivo *toxicity study

Cancer chemotherapeutics would cause systematic toxicity to normal tissue, so we evaluated the *in vivo* toxicity by H&E staining of main organs. No significant pathological changes were recorded in spleen and lung for all groups. However, focal areas of spotty necrosis hepatocytes in clusters were observed in all livers, this may be caused by H22 hepatoma cells. The lobular architecture and overall histological appearance of livers were essentially normal. Free DOX result in slight glomerular atrophy and interstitial fibrosis in kidney. Myofibrillar hypofunction, mild cell necrosis, and inflammation of heart appeared in DOX group, which were also found in CDs-DOX team sparingly. No apparent signs of atrophy, inflammation, cell necrosis, and other nocuous symptoms to heart and kidney were observed in the CDs-ICG-LPs -treated period (Figure S5). Taking all these results into account, it is well illustrated that this multistage CDs-ICG-LPs could effectively reduce systemic toxicity of DOX to main organs. These results could be explained by the reduction of nonspecific toxicity of long circulation and controlled release through photothermal effect.

## 4. Conclusion

A novel multistage nanocarrier named CDs-ICG-LPs was constructed and evaluated. Based on the size switch caused by photothermal effect, the CDs-ICG-LPs delivered the CDs-DOX to the least accessible area of solid tumor, the core in tumor tissues, where tumor stem cells usually stayed, thus enhancing therapeutic efficacy. Besides, ICG’s heating capacity enables their use for hyperthermia, which aims to not only trigger CDs-DOX release from CDs-ICG-LPs complex, but also could be used for accelerating the release of DOX so as to enhance antitumor activity. All these evidences demonstrated that this study provide a promising strategy towards the design of more intelligent nanocarriers for more effective tumor therapy in future.

## Supplementary Material

Supplymentary_Figure-revised.docx

## References

[CIT0001] Bealle G, Di Corato R, Kolosnjaj-Tabi J, et al (2012). Ultra magnetic liposomes for MR imaging, targeting, and hyperthermia. Langmuir 28:11834–42. 22799267 10.1021/la3024716

[CIT0002] Blanco E, Shen H, Ferrari M. (2015). Principles of nanoparticle design for overcoming biological barriers to drug delivery. Nat Biotechnol 33:941–51. 26348965 10.1038/nbt.3330 PMC4978509

[CIT0003] Chen S, Cheng A-C, Wang M-S, Peng X. (2008). Detection of apoptosis induced by new type gosling viral enteritis virus in vitro through fluorescein annexin V-FITC/PI double labeling. World J Gastroenterol 14:2174. 18407590 10.3748/wjg.14.2174 PMC2703841

[CIT0004] Ding H, Du F, Liu P, et al (2015). DNA-carbon dots function as fluorescent vehicles for drug delivery. ACS Appl Mater Interfaces 7:6889–97. 25742297 10.1021/acsami.5b00628

[CIT0005] Duan WR, Garner DS, Williams SD, et al (2003). Comparison of immunohistochemistry for activated caspase-3 and cleaved cytokeratin 18 with the TUNEL method for quantification of apoptosis in histological sections of PC-3 subcutaneous xenografts. J Pathol 199:221–8. 12533835 10.1002/path.1289

[CIT0006] Feng T, Ai X, An G, et al (2016). Charge-convertible carbon dots for imaging-guided drug delivery with enhanced in vivo cancer therapeutic efficiency. ACS Nano 10:4410–20. 26997431 10.1021/acsnano.6b00043

[CIT0007] Feng T, Ai X, Ong H, Zhao Y. (2016). Dual-responsive carbon dots for tumor extracellular microenvironment triggered targeting and enhanced anticancer drug delivery. ACS Appl Mater Interfaces 8:18732. 27367152 10.1021/acsami.6b06695

[CIT0008] Food and Drug Administration. (2013). Product insert: indocyanine green (IC-GreenTM). Available at: http://www.accessdata.fda.gov/drugsatfda_docs/label/2006/011525s017lbl.pdf [last accessed Jan 6].

[CIT0009] Gao Z, Liu X, Wang Y, et al (2016). Facile and one-pot synthesis of Fe_3_O_4_@chitosan nanospheres for MRI and fluorescence imaging guided chemo-photothermal combinational therapy of cancer. Dalton Trans 45:19519. 27897297 10.1039/c6dt03897b

[CIT0010] Gown AM, Willingham MC. (2002). Improved detection of apoptotic cells in archival paraffin sections: immunohistochemistry using antibodies to cleaved caspase 3. J Histochem Cytochem 50:449–54. 11897797 10.1177/002215540205000401

[CIT0011] Hu G, Zhang H, Zhang L, et al (2015). Integrin-mediated active tumor targeting and tumor microenvironment response dendrimer-gelatin nanoparticles for drug delivery and tumor treatment. Int J Pharmaceut 496:1057–68. 10.1016/j.ijpharm.2015.11.025 26598487

[CIT0012] Kobayashi H, Brechbiel MW. (2005). Nano-sized MRI contrast agents with dendrimer cores. Adv Drug Deliv Rev 57:2271–86. 16290152 10.1016/j.addr.2005.09.016

[CIT0013] Kraft JC, Ho RJ. (2014). Interactions of indocyanine green and lipid in enhancing near-infrared fluorescence properties: the basis for near-infrared imaging in vivo. Biochemistry 53:1275–83. 24512123 10.1021/bi500021j PMC3985908

[CIT0014] Li M, Tang Z, Lv S, et al (2014). Cisplatin crosslinked pH-sensitive nanoparticles for efficient delivery of doxorubicin. Biomaterials 35:3851–64. 24495487 10.1016/j.biomaterials.2014.01.018

[CIT0015] Liang R, Wei M, Evans DG, Duan X. (2014). Inorganic nanomaterials for bioimaging, targeted drug delivery and therapeutics. Chem Commun 50:14071–81. 10.1039/c4cc03118k 24955443

[CIT0016] Lim YT, Noh YW, Han JH, et al (2008). Biocompatible polymer-nanoparticle-based bimodal imaging contrast agents for the labeling and tracking of dendritic cells. Small 4:1640–5. 18819168 10.1002/smll.200800582

[CIT0017] Liu K, Liu PC, Liu R, Wu X. (2015). Dual AO/EB staining to detect apoptosis in osteosarcoma cells compared with flow cytometry. Med Sci Monit Basic Res 21:15–20. 25664686 10.12659/MSMBR.893327 PMC4332266

[CIT0018] MacEwan SR, Chilkoti A. (2017). From composition to cure: a systems engineering approach to anticancer drug carriers. Angew Chem Int Ed Engl 56:6712–33. 28028871 10.1002/anie.201610819 PMC6372097

[CIT0019] Maeda H, Tsukigawa K, Fang J. (2016). A Retrospective 30 years after discovery of the enhanced permeability and retention effect of solid tumors: next-generation chemotherapeutics and photodynamic therapy–problems, solutions, and prospects. Microcirculation 23:173–82. 26237291 10.1111/micc.12228

[CIT0020] Maldiney T, Ballet B, Bessodes M, et al (2014). Mesoporous persistent nanophosphors for in vivo optical bioimaging and drug-delivery. Nanoscale 6:13970–6. 25316201 10.1039/c4nr03843f

[CIT0021] Mauceri HJ, Hanna NN, Beckett MA, et al (1998). Combined effects of angiostatin and ionizing radiation in antitumour therapy. Nature 394:287–91. 9685160 10.1038/28412

[CIT0022] Mo R, Sun Q, Li N, Zhang C. (2013). Intracellular delivery and antitumor effects of pH-sensitive liposomes based on zwitterionic oligopeptide lipids. Biomater Sci 34:2773–86. 10.1016/j.biomaterials.2013.01.030 23352118

[CIT0023] Moku G, Gulla SK, Nimmu NV, et al (2016). Delivering anti-cancer drugs with endosomal pH-sensitive anti-cancer liposomes. Biomater Sci 4:627–38. 26806172 10.1039/c5bm00479a

[CIT0024] Nguyen QT, Tsien RY. (2013). Fluorescence-guided surgery with live molecular navigation-a new cutting edge. Nat Rev Cancer 13:653–62. 23924645 10.1038/nrc3566 PMC4427343

[CIT0025] Reina G, Orlanducci S, Cairone C, et al (2015). Rhodamine/nanodiamond as a system model for drug carrier. J Nanosci Nanotechnol 15:1022–9. 26353608 10.1166/jnn.2015.9736

[CIT0026] Saxena V, Sadoqi M, Shao J. (2004). Enhanced photo-stability, thermal-stability and aqueous-stability of indocyanine green in polymeric nanoparticulate systems. J Photochem Photobiol B Biol 74:29–38. 10.1016/j.jphotobiol.2004.01.002 15043844

[CIT0027] Saxena V, Sadoqi M, Shao J. (2003). Degradation kinetics of indocyanine green in aqueo. J Pharmaceut Sci 92:2090–7. 10.1002/jps.10470 14502548

[CIT0028] Sercombe L, Veerati T, Moheimani F, et al (2015). Advances and challenges of liposome assisted drug delivery. Front Pharmacol 6:286. 26648870 10.3389/fphar.2015.00286 PMC4664963

[CIT0029] Shirata C, Kaneko J, Inagaki Y, et al (2017). Near-infrared photothermal/photodynamic therapy with indocyanine green induces apoptosis of hepatocellular carcinoma cells through oxidative stress. Sci Rep 7:13958. 29066756 10.1038/s41598-017-14401-0 PMC5654824

[CIT0030] Sun T-M, Du J-Z, Yao Y-D, et al (2011). Simultaneous delivery of siRNA and paclitaxel via a “Two-in-One” micelleplex promotes synergistic tumor suppression. ACS Nano 5:1483–94. 21204585 10.1021/nn103349h

[CIT0031] Szoka F, Papahadjopoulos D. (1978). Procedure for preparation of liposomes with large internal aqueous space and high capture by reverse-phase evaporation. Biochemistry 75:4194–8. 10.1073/pnas.75.9.4194 PMC336078 279908

[CIT0032] Tang Y, Lei T, Manchanda R, et al (2010). Simultaneous delivery of chemotherapeutic and thermal-optical agents to cancer cells by a polymeric (PLGA) nanocarrier: an in vitro study. Pharm Res 27:2242–53. 20694526 10.1007/s11095-010-0231-6

[CIT0033] Venkatesan R, Pichaimani A, Hari K, et al (2013). Doxorubicin conjugated gold nanorods: a sustained drug delivery carrier for improved anticancer therapy. J Mater Chem B 1:1010–8. 10.1039/c2tb00078d 32262365

[CIT0034] Wang Y, Liu X, Deng G, et al (2017). Multifunctional PS@CS@Au-Fe_3_O_4_-FA nanocomposites for CT, MR and fluorescence imaging guided targeted-photothermal therapy of cancer cells. J Mater Chem B 5:4221. 10.1039/c7tb00642j 32264152

[CIT0035] Wolfbeis OS. (2015). An overview of nanoparticles commonly used in fluorescent bioimaging. Chem Soc Rev 44:4743–68. 25620543 10.1039/c4cs00392f

[CIT0036] Yang L, Jiang W, Qiu L, et al (2015). One pot synthesis of highly luminescent polyethylene glycol anchored carbon dots functionalized with a nuclear localization signal peptide for cell nucleus imaging. Nanoscale 7:6104–13. 25773263 10.1039/c5nr01080b

[CIT0037] Yang P, Wang L, Wang H. (2015). Smart supramolecular nanosystems for bioimaging and drug delivery. Chin J Chem 33:59–70.

[CIT0038] Yasuhiro M, Maeda H. (1986). A new concept for macromolecular therapeutics in cancer chemotherapy: mechanism of tumoritropic accumulation of proteins and the antitumor agent smancs. Cancer Res 46:6387–92. 2946403

[CIT0039] Yuan Y, Guo B, Hao L, et al (2017). Doxorubicin-loaded environmentally friendly carbon dots as a novel drug delivery system for nucleus targeted cancer therapy. Colloids Surfaces B, Biointerfaces 159:349–59. 28806666 10.1016/j.colsurfb.2017.07.030

[CIT0040] Zheng XT, Ananthanarayanan A, Luo KQ, Chen P. (2015). Glowing graphene quantum dots and carbon dots: properties, syntheses, and biological applications. Small 11:1620–36. 25521301 10.1002/smll.201402648

[CIT0041] Zheng M, Liu S, Li J, et al (2014). Integrating oxaliplatin with highly luminescent carbon dots: an unprecedented theranostic agent for personalized medicine. Adv Mater 26:3554–60. 24634004 10.1002/adma.201306192

[CIT0042] Zheng C, Zheng M, Gong P, et al (2012). Indocyanine green-loaded biodegradable tumor targeting nanoprobes for in vitro and in vivo imaging. Biomaterials 33:5603–9. 22575835 10.1016/j.biomaterials.2012.04.044

[CIT0043] Zheng X, Zhou F, Wu B, et al (2012). Enhanced tumor treatment using biofunctional indocyanine green-containing nanostructure by intratumoral or intravenous injection. Mol Pharm 9:514–22. 22332810 10.1021/mp200526m PMC3418867

