# Supplementary material for: Multistage delivery of CDs-DOX/ICG-loaded liposome for highly penetration and effective chemo-photothermal combination therapy
Source: Drug Deliv. 2018 Nov 20;25(1):1826–39. doi: 10.1080/10717544.2018.1482975 (PMC6598495; doi:10.1080/10717544.2018.1482975)
Supplement: Supplymentary_Figure-revised.docx [file IDRD_A_1482975_SM5587.docx]

**Supplymentary Figure**

Tab.S1 The leakage rate of CDs-ICG-LPs under 4℃ or 25℃.

| Time（day） | 5 | 15 | 30 |
| --- | --- | --- | --- |
| 4℃ | 0.56 | 1.01 | 1.17 |
| 25℃ | 3.55 | 10.23 | 23.66 |





Fig.S1.Quantitative evaluation of cell survival for HepG2 cells treated with blank liposomes.





Fig.S2.The cellular uptake of different formulations with incubated for 1, 2, 4h was analyzed by flow cytometry.





Fig.S3.The summary of apoptosis results in HepG2 cells treated with DOX, CDs-DOX or CDs-ICG-LPs for 24 h. (”-” and “+” mean treated without or with laser irradiation).


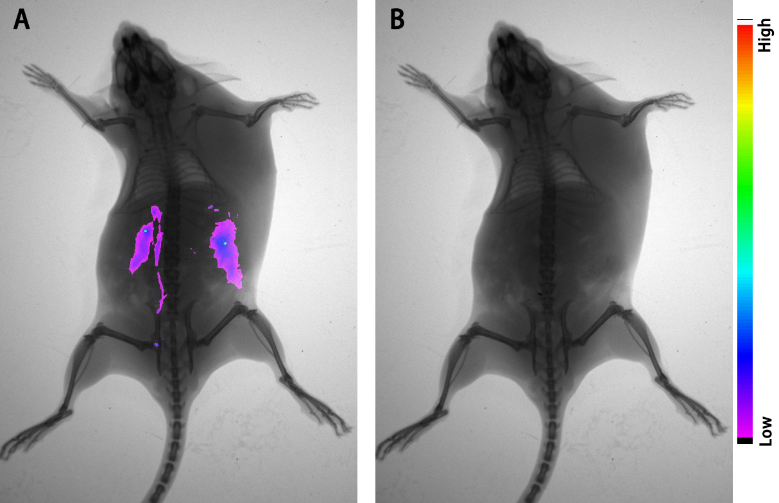


Fig.S4.In vivo imaging of mice before injection under the same conditions as CDs-DOX (A) and ICG (B).


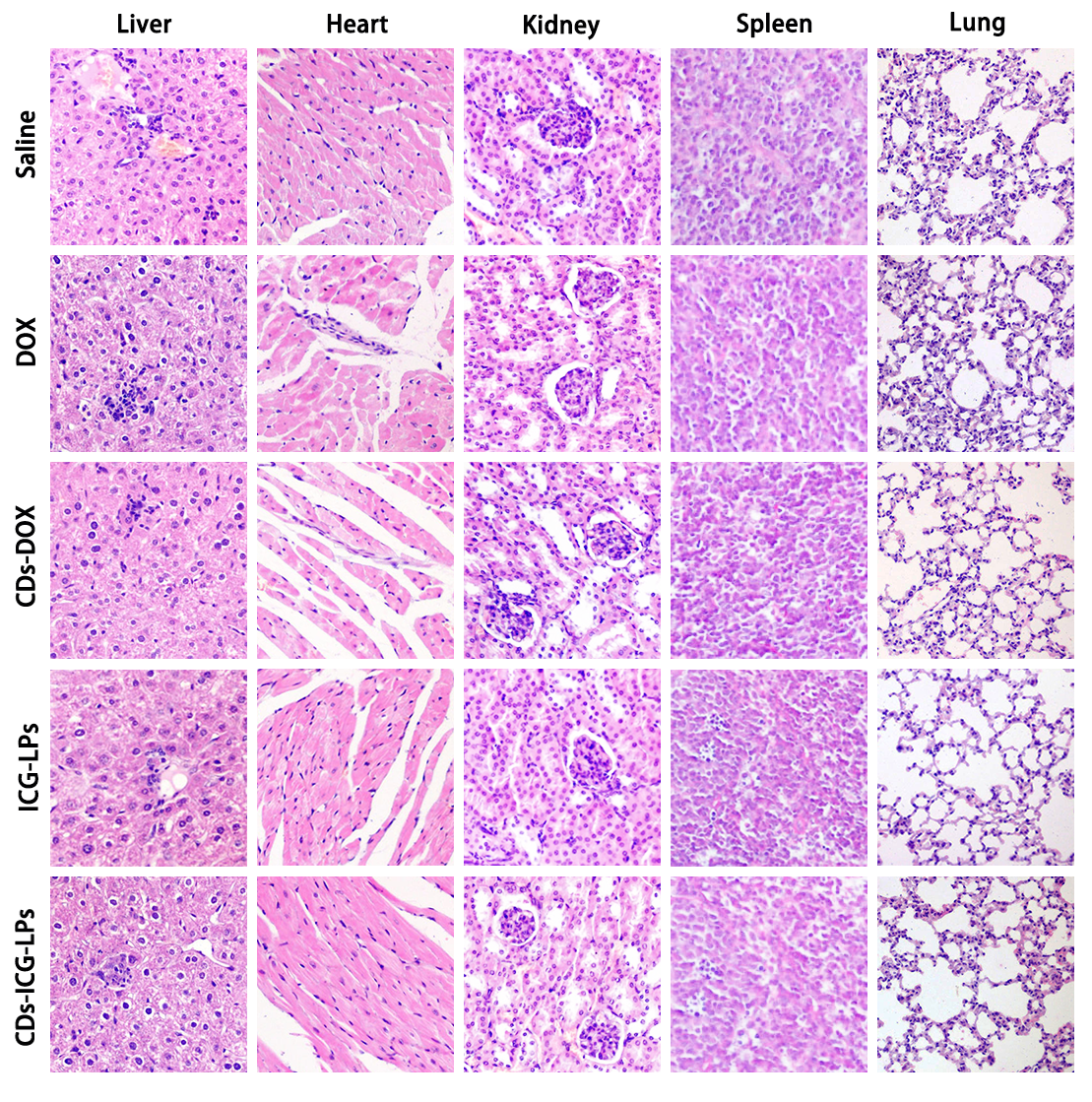


Fig.S5. In vivo toxicity assay of main organs in tumor mice treated with different formulations by H&E staining.
